# Activation of carbonic anhydrases from human brain by amino alcohol oxime ethers: towards human carbonic anhydrase VII selective activators

**DOI:** 10.1080/14756366.2020.1838501

**Published:** 2020-10-25

**Authors:** Alessio Nocentini, Doretta Cuffaro, Lidia Ciccone, Elisabetta Orlandini, Susanna Nencetti, Elisa Nuti, Armando Rossello, Claudiu T. Supuran

**Affiliations:** a Section of Pharmaceutical and Nutraceutical Sciences, Department of Neuroscience, Psychology, Drug Research and Child’s Health (Neurofarba), University of Florence, Sesto Fiorentino, Italy; b Department of Pharmacy, University of Pisa, Pisa, Italy

**Keywords:** Metalloenzyme, activation, selectivity, CNS-disease, cognition

## Abstract

The synthesis and carbonic anhydrase (CA; EC 4.2.1.1) activating effects of a series of oxime ether-based amino alcohols towards four human (h) CA isoforms expressed in human brain, hCA I, II, IV and VII, are described. Most investigated amino alcohol derivatives induced a consistent activation of the tested CAs, with K_A_s spanning from a low micromolar to a medium nanomolar range. Specifically, hCA II and VII, putative main CA targets when central nervous system (CNS) diseases are concerned, were most efficiently activated by these oxime ether derivatives. Furthermore, a multitude of selective hCA VII activators were identified. As hCA VII is one of the key isoforms involved in brain metabolism and other brain functions, the identified potent and selective hCA VII activators may be considered of interest for investigations of various therapeutic applications or as lead compounds in search of even more potent and selective CA activators.

## 1. Introduction

Activators of the metalloenzymes carbonic anhydrases (CAs; EC 4.2.1.1, CAAs) have been lately going through a second youth in drug discovery processes^1^. Early evidence of the CA activation efficacy of amines, such as histamine, dating back to the 1940s was thereafter long debated up, and even considered as an experimental artefact^2^^,^^3^. In the early 1990s, the combination of highly purified enzymes and precise techniques, such as the stopped flow assay, put an end to the long controversy, testifying the undeniable existence of CAAs^4–7^. CAAs intervene in the second and rate determining step of the catalytic mechanism of carbon dioxide reversible hydration (Equations 1 and 2), that is the regeneration of the catalytically active, zinc hydroxide species, by a proton transfer reaction from the Zn^2+^ – bound water molecule to the external medium (Equation 2)^6–8^. This process is assisted by active site residues acting as proton shuttle, such as His residues placed in the middle or at the entrance of the active site cavity of the α-class human CAs^4^.

(1) $${\text{EZn}}^{2+}-{\text{OH}}^{-}+{\;\text{CO}}_{2}\;⇄\;{\text{EZn}}^{2+}-{\text{HCO}}_{3}^{-}+{\text{ H}}_{2}O\;⇄\;{\text{EZn}}^{2+}-{\text{OH}}_{2}+{\;\text{HCO}}_{3}^{-}$$

(2) $${\text{EZn}}^{2+}-{\text{OH}}_{2}\;⇄\;{\text{EZn}}^{2+}-{\text{OH}}^{-}+{\text{ H}}^{+}$$

(3) $${\text{EZn}}^{2}{}^{+}-{\text{OH}}_{2}+\;A\;⇄\;[{\text{EZn}}^{2}{}^{+}-{\text{OH}}_{2}-A]\;⇄\;[{\text{EZn}}^{2}{}^{+}-{\text{OH}}^{-}-{\text{AH}}^{+}]\;⇄\;{\text{EZn}}^{2}{}^{+}-{\text{OH}}^{-}+{\text{AH}}^{+}$$

The activators non-competitively activate the CAs through the formation of a ternary complex consisting of the enzyme, the substrate, and the activator. CAAs bind apart from the metal-coordination system, namely at the middle edge of CAs active site cavity, where they assist the proton shuttling^9–11^. As a proof of this phenomenon, it was demonstrated that efficient activators possess p*K*_a_ values in the range of 6–8, values similar to the p*K*_a_ of the His imidazole moiety^7^.

It is seemingly odd that CAs, among the most efficient enzymes in Nature, may be activated for biomedical purposes^8^. However, genetic deficiencies of several CA isoforms (e.g. human CA I, II, IV, VA, XII and XIV) have been reported in the last decades, associated to diseases such as osteopetrosis, cerebral calcifications, retinal problems, hyperammonaemia, hyperchlorhydrosis^12–15^, and the loss of function of these enzymes would in principle be treatable with CA selective activators^8^. In addition, there is evidence that CAs activation improves memory deficits, cognitive performance and learning^16–18^, being nine of the fifteen known human CA isoforms present in brain^19^^,^^20^. Contrariwise, other evidence supported that CA inhibitors (CAIs) impair memory in human, according to studies on the CAIs topiramate and acetazolamide during acute high-altitude exposure^21^^,^^22^. Thus, CAs represent a crucial family of new targets for improving cognition, but also in therapeutic areas, such as phobias, obsessive-compulsive disorder, generalised anxiety, and post-traumatic stress disorders, for which few effective therapies are available. In fact, in a recent paper, one of our groups showed that the CAA D-phenylalanine and the CAI acetazolamide are respectively able to reinforce and impair extinction memory, that is, a new memory trace that inhibits the expression of the memory of a traumatic event^23^. In this golden period for CAAs, the discovery of new brain isoform selective CAAs (as well as CAIs) is invaluable to elucidate the role of CA isoforms in brain processes. In addition, CAAs are considered relevant in artificial tissues and in CO_2_ capture and sequestration processes^1^^,^^24^.

Here, we extend the knowledge of CAA chemotypes by describing the synthesis and CA activating effects of a series of oxime ether based amino alcohols towards four hCA isoforms expressed in human brain.

## 2. Material and methods

### 2.1. Chemistry

^1^H and ^13 ^C NMR spectra were recorded on a Bruker Avance III HD 400 MHz spectrometer. Chemical shifts (δ) are reported in parts per million and coupling constants (*J*) are reported in hertz (Hz). ^13 ^C NMR spectra were fully decoupled. The following abbreviations were used to explain multiplicities: singlet (s), doublet (d), triplet (t), double doublet (dd), broad (br), and multiplet (m). Chromatographic separations were performed on silica gel columns by flash column chromatography (Kieselgel 40, 0.040−0.063 mm, Merck). Reactions were followed by thin-layer chromatography (TLC) on Merck aluminium silica gel (60 F254) sheets that were visualised under a UV lamp. Evaporation was performed *in vacuo* (rotating evaporator). Sodium sulphate was always used as the drying agent. Commercially available chemicals were purchased from Sigma-Aldrich.

#### 2.1.1. General procedure for the synthesis of amino alcohols 1–13

A solution of the proper oxime (**17**–**27**) (10 mmol) in anhydrous DMF (10 ml) was added portionwise to a solution of MeONa in MeOH (30 ml), prepared from anhydrous MeOH (30 ml) and Na (11 mmol). The reaction mixture was stirred at 60 °C for 1 h and then cooled at rt. Epichlorohydrin (0.86 ml, 11 mmol) dissolved in anhydrous DMF (10 ml) was added dropwise, and the resulting mixture was stirred for 1 h at rt, poured into water (100 ml) and extracted with CHCl_3_ (2 × 100mL). The organic phases were combined, washed with water (2 × 200 ml), dried (Na_2_SO_4_) filtered and evaporated under reduced pressure. The crude was distilled *in vacuo* affording an oil corresponding to the proper epoxide (**28**–**38**, yield 75–85%). NMR data of **28**–**35** were in accordance with those reported in literature^25–33^.

*(E)-4-Chlorobenzaldehyde O-oxiran-2-ylmethyl oxime (****36****)*: yield 76%; ^1^H NMR (400 MHz, CDCl_3_): δ 8.40 (s, 1H), 7.35 (m, 2H), 7.30 (m, 2H), 4.38 (dd, *J* = 12.3 Hz, *J* = 3.5 Hz, 1H), 4.13 (dd, *J* = 12.3 Hz, *J* = 3.5 Hz, 1H), 3.28–3.32 (m, 1H), 2.88 (dd, *J* = 5.2 Hz, *J* = 4.2 Hz, 1H), 2.70 (dd, *J* = 5.2 Hz, *J* = 4.2 Hz, 1H).

*(E)-2-Chlorobenzaldehyde O-oxiran-2-ylmethyl oxime (****37****)*: yield 82%; ^1^H NMR (400 MHz, CDCl_3_): δ 8.34 (s, 1H), 7.37 (m, 2H), 7.28–7.24 (m, 2H), 4.35 (dd, *J* = 12.3 Hz, *J* = 3.5 Hz, 1H), 4.06 (dd, *J* = 12.3 Hz, *J* = 3.5 Hz, 1H), 3.30–3.34 (m, 1H), 2.90 (dd, *J* = 5.2 Hz, *J* = 4.2 Hz, 1H), 2.76 (dd, *J* = 5.0 Hz, *J* = 4.2 Hz, 1H).

*(E)-3-Chlorobenzaldehyde O-oxiran-2-ylmethyl oxime (****38****)*: yield 79%; ^1^H NMR (400 MHz, CDCl_3_): δ 8.51 (s, 1H), 7.34–7.28 (m, 1H), 7.26–7.23 (m, 3H), 4.32 (dd, *J* = 12.1 Hz, *J* = 3.7 Hz, 1H), 4.18 (dd, *J* = 12.1 Hz, *J* = 3.7 Hz, 1H), 3.31–3.35 (m, 1H), 2.88 (dd, *J* = 5.0 Hz, *J* = 4.1 Hz, 1H), 2.67 (dd, *J* = 5.0 Hz, *J* = 4.1 Hz, 1H).

A stirred solution of epoxide (**28**–**38**, 10 mmol) in dry benzene (6 ml) was treated with an excess of isopropylamine or *tert*-butylamine (50 mmol). The reaction mixture was stirred for 12 h at 90 °C, and then evaporated. The crude was dissolved in a mixture of MeOH/EtOH 3:7 (20 ml) and treated with 1.2 equivalent of the proper organic acid (oxalic, malic or fumaric acids) to give the corresponding amino alcohol (**1**–**13**) as a white solid salt.

*(E)-2,3-Dihydro-1H-inden-1-one O-(3-(tert-butylamino)-2-hydroxypropyl) oxime maleate* **1**: Maleate salt. ^1^H NMR (400 MHz, CDCl_3_): δ 9.51 (s, 1H), 7.94 (s, 1H), 7.61 (d, *J* = 7.6 Hz, 1H), 7.34–7.31(m, 2H), 7.26–7.23 (m, 1H), 6.18 (s, 2H), 4.43–4.38 (m, 1H), 4.29 (dd, *J* = 4.08 Hz, *J* = 12 Hz, 1H), 4.19 (dd, *J* = 6.0 Hz, *J* = 11.6 Hz, 1H), 3.21–3.28 (m, 1H); 3.05–3.02 (m, 3H), 2.90–2.87 (m, 2H), 1.43 (s, 9H). ^13 ^C NMR (100 MHz, DMSO-d_6_): δ 167.7, 163.5, 148.9, 136.6, 135.8, 131.1, 127.5, 126.4, 121.4, 75.7, 66.1, 56.8, 44.4, 28.5, 26.8, 25.45.

*(E)-Benzaldehyde O-(3-(tert-butylamino)-2-hydroxypropyl) oxime maleate* **2**: Maleate salt. ^1^H NMR (400 MHz, DMSO-d_6_): δ 8.32 (s, 1H), 7.65–7.62 (m, 2H), 7.45–7.44 (m, 3H), 6.02 (s, 2H), 4.14–4.13 (m, 2H), 3.70–3.67 (m, 1H), 3.11–3.08 (m, 1H), 2.88–2.83 (m, 1H), 1.28 (s, 9H). ^13 ^C NMR (100 MHz, DMSO-d_6_): δ 167.2, 149.5, 136.1, 131.7, 130.1, 128.8, 126.9, 75.4, 65.5, 56.4, 43.9, 25.0.

*Propan-2-one O-(3-(tert-butylamino)-2-hydroxypropyl) oxime oxalate* ***3***: oxalate salt. ^1^H NMR (400 MHz, DMSO-d_6_): δ 4.00–3.87 (m, 3H), 2.99 (dd, *J* = 2.4 Hz, *J* = 12.4 Hz, 1H), 2.76 (dd, *J* = 9.1 Hz, *J* = 12.4 Hz, 1H), 1.81 (s, 3H), 1.26 (s, 9H). ^13 ^C NMR (100 MHz, DMSO-d_6_): δ 164.7, 155.0, 74.4, 65.5, 56.0, 44.1, 25.0, 21.2.

*(E)-4-Methoxybenzaldehyde O-(3-(tert-butylamino)-2-hydroxypropyl) oxime maleate* **4**: Maleate salt. ^1^H NMR (400 MHz, DMSO-d_6_): δ 8.24 (s, 1H), 7.58 (m, 2H), 7.02 (m, 2H), 6.01 (s, 2H), 4.27–4.07 (m, 6H), 3.33 (s, 3H), 3.15–3.05 (m, 1H), 2.94–2.89 (m, 1H), 1.27 (s, 9H). ^13 ^C NMR (100 MHz, DMSO-d_6_): δ 165.4, 161.2, 149.4, 128.9, 124.7, 114.8, 75.8, 65.9, 56.5, 55.7, 44.6, 25.4.

*(E)-3-Methoxybenzaldehyde O-(3-(tert-butylamino)-2-hydroxypropyl) oxime oxalate* **5**: Oxalate salt. ^1^H NMR (400 MHz, DMSO-d_6_): δ 8.27 (s, 1H), 7.35 (t, *J* = 7.6 Hz, 2H), 7.21–7.17 (m, 2H), 7.01 (dd, *J* = 1.6 Hz, *J* = 8 Hz, 1H), 4.12–4.05 (m, 3H), 3.77 (s, 3H), 3.09–3.06 (m, 1H), 2.86–2.81 (m, 1H), 1.28 (s, 9H). ^13 ^C NMR (100 MHz, DMSO-d_6_): δ 165.4, 159.9, 149.8, 133.6, 130.4, 119.9, 116.5, 112.1, 76.1, 65.9, 56.5, 55.6, 44.6, 25.4.

*(E)-2-Methoxybenzaldehyde O-(3-(tert-butylamino)-2-hydroxypropyl) oxime oxalate* ***6***: Oxalate salt. ^1^H NMR (400 MHz, DMSO-d_6_): δ 8.42 (s, 1H), 7.65 (dd, *J* = 1.6 Hz, *J* = 8 Hz, 2H), 7.43 (m, 1H), 7.10 (d, *J* = 8 Hz, 1H), 6.99 (t, *J* = 7.2 Hz, 1H), 4.11–4.05 (m, 3H), 3.83 (s, 3H), 3.07 (bd, *J* = 12 Hz, 1H), 2.86–2.83 (m, 1H), 1.28 (s, 9H). ^13 ^C NMR (100 MHz, DMSO-d_6_): δ 165.2, 157.7, 145.2, 132.2, 126.3, 121.1, 120.0, 112.4, 76.0, 65.6, 56.2, 50.2, 47.3, 19.2.

*(E)-3-Chlorobenzaldehyde O-(3-(tert-butylamino)-2-hydroxypropyl) oxime oxalate* **7**: Oxalate salt. ^1^H NMR (400 MHz, DMSO-d_6_): δ 8.32 (s, 1H), 7.68 (m, 1H), 7.60–7.59 (m, 1H), 7.50–7.47 (m, 2H), 4.15–4.05 (m, 3H), 3.08–3.05 (bd, *J* = 12 Hz, 1H), 2.83 (dd, *J* = 8.8 Hz, *J* = 12.4, 1H), 1.27 (s, 9H). ^13 ^C NMR (100 MHz, DMSO-d_6_): δ 165.4, 148.6, 134.4, 131.3, 130.3, 126.9, 125.9, 76.3, 65.9, 56.5, 44.6, 25.5.

*(E)-4-Chlorobenzaldehyde O-(3-(tert-butylamino)-2-hydroxypropyl) oxime* oxalate **8**: Oxalate salt. ^1^H NMR (400 MHz, DMSO-d_6_): δ 8.32 (s, 1H), 7.68 (m, 1H), 7.65 (m, 2H), 7.51 (m, 2H), 4.14–4.09 (m, 3H), 3.09–3.05 (bd, *J* = 12.4 Hz, 1H), 2.83 (m, 1H), 1.27 (s, 9H). ^13 ^C NMR (100 MHz, DMSO-d_6_): δ 165.4, 148.6, 134.4, 131.2, 130.2, 126.9, 125.9, 76.3, 65.9, 56.5, 44.6, 25.4.

*Cyclohexanone O-(2-hydroxy-3-(isopropylamino)propyl) oxime* oxalate **9**: Oxalate salt. ^1^H NMR (400 MHz, DMSO-d_6_): δ 3.90–3.83 (m, 3H), 3.01–2.98 (m, 1H), 2.80–2.77 (m, 1H), 2.67–2.63 (m, 1H), 2.40–2.38 (m, 2H), 2.12 (t, *J* = 6.8 Hz, 2H), 1.61–1.48 (m, 6H), 1.08 (d, *J* = 6.4 Hz, 6H). ^13 ^C NMR (100 MHz, DMSO-d_6_): δ 165.35, 160.0, 75.5, 67.0, 49.4, 31.8, 27.0, 25.7, 25.2.

*(E)-1,7,7-Trimethylbicyclo[2.2.1]heptan-2-one O-(2-hydroxy-3-(isopropylamino)propyl) oxime fumarate* **10**: Fumarate salt. ^1^H NMR (400 MHz, DMSO-d_6_): δ 6.47 (s, 1H), 4.00–3.91 (m, 2H), 3.87–3.82 (m, 1H), 3.24–3.21 (m, 1H), 2.96–2.93 (m, 1H), 2.78–2.76 (m, 1H), 2.50–2.40 (m, 2H), 1.93 (dd, *J* = 2.4 Hz, *J* = 17.6 Hz, 1H), 1.86 (t, *J* = 3.2 Hz, 1H), 1.78–1.74 (m, 1H), 1.71–1.64 (m, 1H), 1.32–1.28 (m, 1H), 1.18 (t, *J* = 6.0 Hz, 6H), 0.91 (s, 3H), 0.87 (s, 3H), 0.71 (s, 3H). ^13 ^C NMR (100 MHz, DMSO-d_6_): δ 169.2, 168.3, 135.5, 75.3, 65.9, 51.8, 49.7, 48.2, 43.5, 33.9, 27.2, 19.6, 19.5, 18.9, 18.7, 11.6.

*Propan-2-one O-(2-hydroxy-3-(isopropylamino)propyl) oxime* oxalate **11**: Oxalate salt. ^1^H NMR (400 MHz, DMSO-d_6_): δ 4.04–3.99 (m, 1H), 3.94 (dd, *J* = 5.36 Hz, *J* = 11.2 Hz, 1H), 3.86 (dd, *J* = 5.32 Hz, *J* = 11.2 Hz, 1H), 3.34–3.29 (m, 2H), 3.00 (dd, *J* = 2.64 Hz, *J* = 12.56 Hz, 1H), 2.85–2.80 (m, 1H), 1.80 (s, 6H), 1.21 (t, *J* = 6.04 Hz, 6H). ^13 ^C NMR (100 MHz, DMSO-d_6_): δ 165.4, 155.4, 75.0, 66.0, 50.1, 47.5, 21.7, 19.2, 18.5.

*(E)-4-Chlorobenzaldehyde O-(2-hydroxy-3-(isopropylamino)propyl) oxime oxalate* **12**: Oxalate salt. ^1^H NMR (400 MHz, DMSO-d_6_): δ 8.31 (s, 1H), 7.65 (m, 2H), 7.51 (m, 2H), 4.13–4.10 (m, 3H), 3.33–3.28 (m, 1H), 3.08–3.04 (m, 1H), 2.91–2.86 (m, 1H), 1.21 (t, *J* = 6.4 Hz, 6H). ^13 ^C NMR (100 MHz, DMSO-d_6_): δ 165.5, 148.6, 134.4, 134.0, 131.2, 130.2, 127.7, 125.9, 76.4, 65.6, 50.1, 47.3, 19.24, 18.7.

*(E)-2-Chlorobenzaldehyde O-(2-hydroxy-3-(isopropylamino)propyl) oxime* oxalate **13**: Oxalate salt. ^1^H NMR (400 MHz, DMSO-d_6_): δ 8.49 (s, 1H), 7.81 (dd, *J* = 1.6 Hz, *J* = 7.6 Hz, 1H), 7.55 (dd, *J* = 1.2 Hz, *J* = 8 Hz, 1H), 7.48 (dt, *J* = 1.6 Hz, *J* = 7.2 Hz, 1H), 7.41 (m, 1H), 4.16–4.11 (m, 3H), 3.34–3.31 (m, 1H), 3.10–3.07 (m, 1H), 2.93–2.88 (m, 1H), 1.22 (t, *J* = 6.4 Hz, 6H). ^13 ^C NMR (100 MHz, DMSO-d_6_): δ 165.25, 146.2, 133.2, 132.2, 130.4, 129.6, 128.1, 127.7, 76.5, 65.6, 50.2, 47.2, 19.2, 18.6.

#### 2.1.2. General procedure for the synthesis of aminoalcohols 14–16

Epichlorohydrin (0.88 ml,11.2 mmol) was added dropwise to a stirred solution of *N-*hydroxy-5-norbornene-2,3-dicarboximide (2 g, 11.2 mmol) and Et_3_N (3.12 ml, 22.4 mmol) in anhydrous DMF (8 ml). After stirring for 18 h at rt the reaction mixture was poured into water (50 ml) and extracted with CHCl_3_ (2×50mL). The organic phases were combined, washed with water (2 × 100 ml), dried (Na_2_SO_4_) filtered and evaporated under reduced pressure. The crude epoxide **39** was purified by crystallisation with *n*-hexane. NMR data were in accordance with those reported in literature^25^.

A stirred solution of epoxide **39** (611 mg, 2.6 mmol) in dry EtOH (8 ml) was treated with an excess of iPrNH_2_ (0.43 ml, 5 mmol) or t-BuNH_2_ (0.52 ml, 5 mmol). The reaction mixture was stirred at 50 °C for 4 h, and then the solvent was evaporated. The crude was constituted principally by the amino alcohol **40** (iPr) or **41**(tBu) and it was used in the next reaction step without further purification.

A solution of the proper amino alcohol **40** or **41** (1.56 mmol) in 7.8 ml of NH_3_-MeOH 7 N was stirred rt for 2 h. The resulting mixture was filtered and evaporated. The crude was purified by flash chromatography with EtOAc/MeOH/Et_3_N (8: 1.5: 0.5) affording derivative **42** or **43** (60–63% yield). NMR data were in accordance with those reported in literature^25^.

A solution of *p*-, *m*-, or *o*- hydroxybenzaldehyde (6.75 mmol) in EtOH (11.4 ml) and the proper oxyamine **42** or **43** (6.75 mmol) was refluxed for 12 h. After cooling, the mixture was evaporated. The crude was dissolved in a mixture of MeOH/EtOH 3:7 (20 ml) and treated with 1.2 equivalent of the proper organic acid (oxalic, malic or fumaric acids) affording the amino alcohols (**14**–**16**, 70–80% yields) as white solids after crystallisation from MeOH/Et_2_O.

*(E)-2-Hydroxybenzaldehyde O-(2-hydroxy-3-(isopropylamino)propyl) oxime maleate* **14**: Maleate salt. ^1^H NMR (400 MHz, DMSO-d_6_): δ 9.99(bs, 1H), 8.43 (s, 1H), 8.29 (bs, 1H), 7.54 (d, *J* = 7.6 Hz, 2H), 7.27 (m, 1H), 6.90 (d, *J* = 7.8 Hz, 1H), 6.85 (t, *J* = 7.6 Hz, 1H), 6.01 (s, 2H), 5.76 (m, 1H), 4.12–4.07 (m, 3H), 3.33–3.30 (m, 1H), 3.09–3.07 (m, 1H), 2.90–2.89 (m, 1H), 1.22 (m, 6H). ^13 ^C NMR (100 MHz, DMSO-d_6_): δ 167.6, 156.5, 147.4, 132.0, 127.4, 119.9, 117.8, 116.7, 75.9, 65.7, 50.3, 47.1, 19.3, 18.5.

*(E)-3-Hydroxybenzaldehyde O-(3-(tert-butylamino)-2-hydroxypropyl) oxime fumarate* **15**: Fumarate salt. ^1^H NMR (400 MHz, DMSO-d_6_): δ 8.17 (s, 1H), 7.21 (t, *J* = 7.6 Hz, 1H), 7.04–7.00 (m, 2H), 6.82–6.80 (m, 2H), 6.40 (s, 1H), 4.07–4.06 (m, 2H), 4.02–3.94 (m, 1H), 2.89–2.86 (m, 1H), 2.71–2.66 (m, 1H), 1.18 (s, 9H). ^13 ^C NMR (100 MHz, DMSO-d_6_): δ 169.8, 158.2, 149.7, 133.5, 130.3, 118.6, 117.7, 113.3, 76.4, 66.6, 54.5, 45.0, 26.5.

*(E)-4-Hydroxybenzaldehyde O-(3-(tert-butylamino)-2-hydroxypropyl) oxime fumarate* **16**: ^1^H NMR (400 MHz, DMSO-d_6_): δ 8.12 (s, 1H), 7.42 (m, 2H), 6.80 (m, 2H), 6.40 (s, 1H), 4.02–3.97 (m, 3H), 3.04–3.00 (m, 1H), 2.87–2.83 (m, 1H), 2.70–2.65 (m, 1H), 1.10 (d, *J* = 6.4 Hz, 6H).^13^C NMR (100 MHz, DMSO-d_6_): δ 169.0, 159.8, 149.3, 129.0, 123.1, 116.1, 76.4, 66.9, 49.4, 48.9, 21.1, 20.7.

### 2.2. Carbonic anhydrase activation

A stopped-flow method^34^ has been used for assaying the CA catalysed CO_2_ hydration activity with Phenol red as indicator, working at the absorbance maximum of 557 nm, following the initial rates of the CA-catalysed CO_2_ hydration reaction for 10–100 s. For each activator, at least six traces of the initial 5–10% of the reaction have been used for determining the initial velocity. The uncatalyzed rates were determined in the same manner and subtracted from the total observed rates. Stock solutions of activator (0.1 mM) were prepared in distilled-deionised water and dilutions up to 0.1 nM were done thereafter with the assay buffer. The activation constant (K_A_), defined similarly with the inhibition constant (K_I_), was obtained by considering the classical Michaelis–Menten equation (Equation 4), which has been fitted by nonlinear least squares by using PRISM 3:

(4) $$v={\;v}_{\text{max}}/\left\{1\;+{\;K}_{M}/\left[S\right]\;\left(1\;+\;{\left[A\right]}_{f}/K_{A}\right)\right\}$$

where [A]_f_ is the free concentration of activator.

Working at substrate concentrations considerably lower than K_M_ ([S]≪K_M_), and considering that [A]_f_ can be represented in the form of the total concentration of the enzyme ([E]_t_) and activator ([A]_t_), the obtained competitive steady-state equation for determining the activation constant is given by Equation (5):

(5) $$v\;=v_{0}K_{A}/\{K_{A}+({\left[A\right]}_{t}–0.5\{\left({\left[A\right]}_{t}+{\left[E\right]}_{t}+{\text{K}}_{A}\right)–{\left({\left[A\right]}_{t}+{\left[E\right]}_{t}+{\text{K}}_{A}\right)}^{2}–4{\left[A\right]}_{t}{\left[E\right]}_{t}^{1/2}\}\}$$

where v_0_ represents the initial velocity of the enzyme-catalysed reaction in the absence of an activator^35–37^. Enzyme concentrations in the assay system were in the range of 9–12 nM.

## 3. Results and discussion

### 3.1. Chemistry

**Figure 1.**
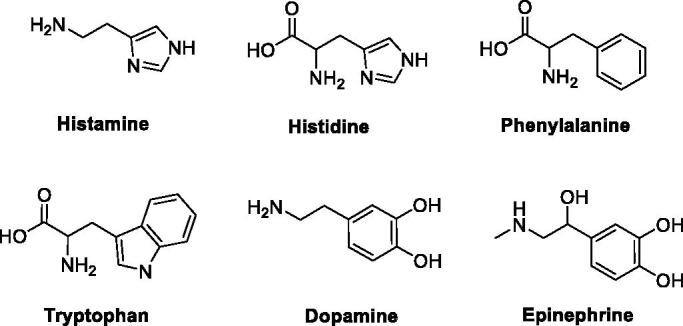
Natural amino acids and amines investigated for the activation of catalytically active hCA isoforms.

**Figure 2.**
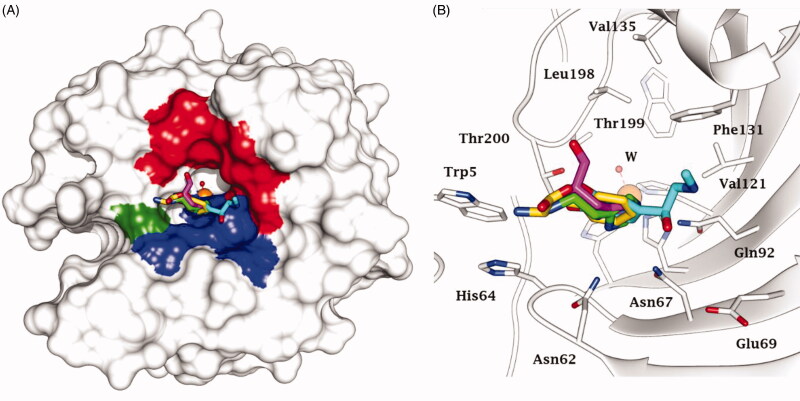
Superimposition of CAA – hCA II complexes as determined by X-ray crystallography. (A) Surface view: the hydrophobic half of the active site is coloured red; His^64^ is coloured green and the hydrophilic half in blue. (B) Ribbon active site view. The activators are histamine, in green (PDB 1AVN); L-His, in magenta (PDB 2ABE); L-Phe, in gold (PDB 2FMG); adrenaline, in cyan (PDB 2HKK).

**Figure 3.**
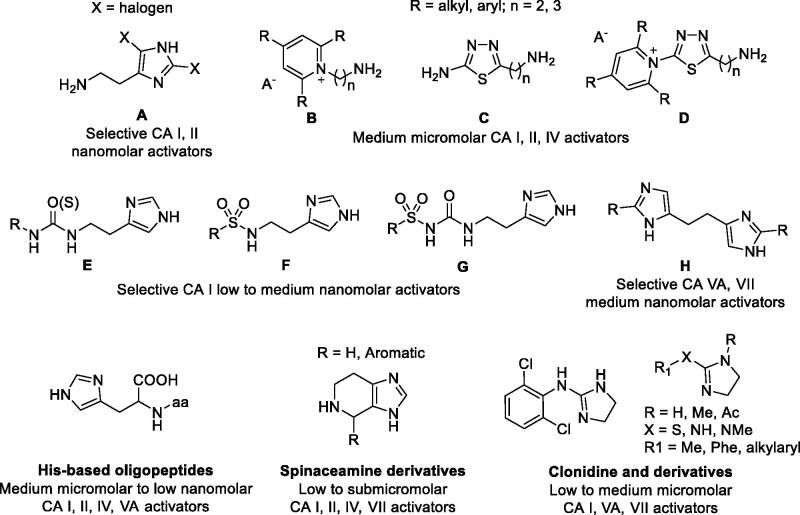
Histamine-based carbonic anhydrase activators.

As mimic of the main proton shuttling residue (i.e. histidine), histamine (Figure 1) is the main lead for designing CAAs^8^. The binding mode of histamine with hCA II was elucidated by X-ray crystallography (Figure 2)^6^. In the CAA/enzyme adduct, a complex network of H-bonds involve the Zn-bound water molecule, His64 and the imidazole ring of the activator, located far away from the metal ion (Figure 2). Successive X-ray crystallographic studies showed that many other amines and amino acids (Figure 1) bind in this area and share flexible tails decorated with protonable moieties (Figure 2)^38–40^. No isoform-selective CAAs were detected so far among these amines and amino acid derivatives, except for few exceptions (e.g. histamine shows a 10 nM K_A_s against hCAs VA and XIV but is a micromolar activator of the remaining isoforms)^1^. The molecule of histamine has been extensively modified (Figure 3), including substituents on the imidazole C atoms (**A**)^41^^,^^42^, replacing the imidazole ring with other heterocycles, such as 2,4,6-trisubstituted pyridinium (**B**), 1,3,4-thiadiazole (**C**) or a combination of these two ring systems (**D**)^43^^,^^44^ and functionalising the NH_2_ group, as in carboxamides/ureas/thioureas (**E**)^45^, sulphonamides (**F**)^46^, arylsulfonylureas (**G**)^47^, bis-histamine (**H**)^48^^,^^49^, oligopeptides^49^^,^^50^, or imidazole/imidazoline derivatives of the alkaloyd spinaceamine^51^^,^^52^ and drug clonidine^53^ (Figure 3) were reported to possess improved CA activatory profile when potency and isoform selectivity are concerned.

**Figure 4.**
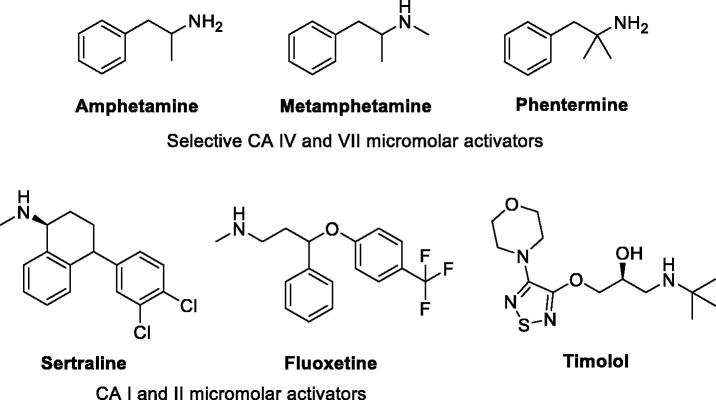
Other pharmacological agents showing hCAs activating properties.

Interestingly, pharmacological agents born for other medical applications (Figure 4) showed also to possess more or less relevant CA-activating effects, among which psychoactive compounds of the amphetamine and methamphetamine family^54^, the selective serotonin reuptake inhibitors fluoxetine, sertraline and citalopram^55^, the phosphodiesterase IV inhibitor sildenafil^56^, and the β-blocker amino alcohol derivative timolol^57^. Timolol was taken as lead compound in the present study to design a new series of uncommon CAAs, which do not bear imidazole like scaffolds, intesively explored in the field. The enzyme kinetic method showed that timolol noncompetitively activates hCA I and II by forming of a ternary complex consisting of the enzyme, the substrate, and timolol. Docking studies were used to point out that timolol also binds at the entrance of the active site cavity nearby the proton shuttle residue His64^57^. Thus, a series of aminoalcohol oxime ether derivatives previously shown to possess β-blocking or analgesic/antiarrhythmic activity (compounds **1**–**16**)^25–32^, was investigated for the activation of a panel of brain hCAs.

**Scheme 1.**
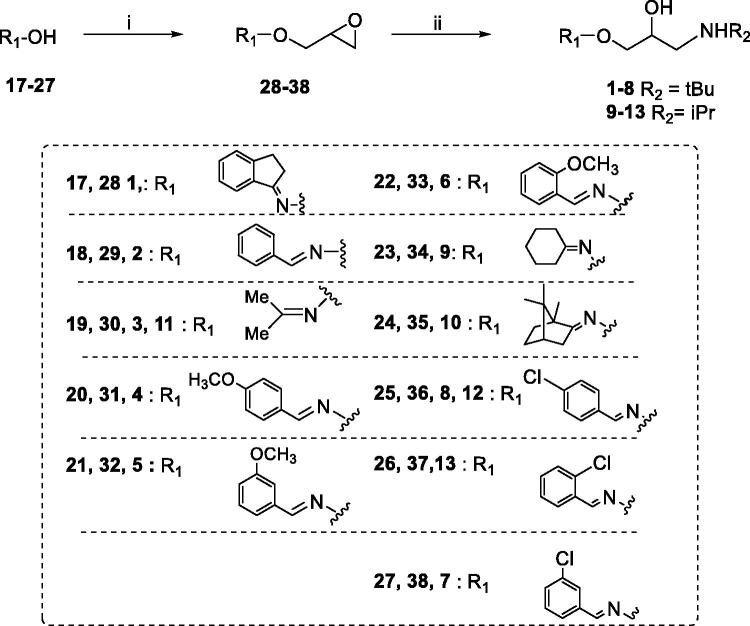
Preparation of Amino alcohols **1–13**. Reagents and conditions: (i) epichlorohydrin, MeONa/MeOH, dry DMF, 60 °C, 1 h; (ii) *i*PrNH_2_ or *t*BuNH_2_, Benzene, 90 °C, 12 h.

**Scheme 2.**
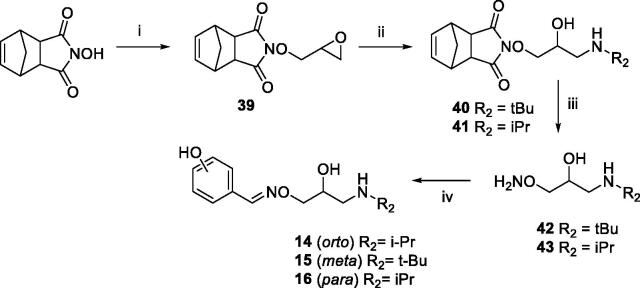
Preparation of Amino alcohols **14–16**. Reagents and conditions: (i) epichlorohydrin, Et_3_N, dry DMF, rt, 18 h; (ii) *i*PrNH_2_ or *t*BuNH_2_, Benzene, 50 °C, 4 h; (iii) NH_3_ MeOH 7 N, rt, 2 h; (iv) *o*-, *m*-, *p*-hydroxybenzaldehyde, EtOH, 90 °C, 12 h.

Compounds **1**–**6** and **9**–**16** were prepared as previously described (Schemes 1 and 2)^25–32^. Here, an updated synthetic procedure is reported. Moreover, new ^1^H NMR and ^13 ^C NMR spectra of final compounds are provided in the experimental section. A fully detailed description is provided for the synthesis of new compounds **7** and **8**, following the same synthetic procedure.

Amino alcohols **1–13** were synthesised as reported in Scheme 1^26–32^. Oximes **17–27** were treated with epichlorohydrin in strong basic conditions affording epoxides **28–38**. The amino alcohols **1–13** were obtained by reaction of oxime ethers **28–38** with an excess of the proper amine, *i*PrNH_2_ or *t*BuNH_2_. The final compounds were purified by crystallisation as organic salts.

The phenolic derivatives **14**–**16** were prepared as described in Scheme 2. *N-*hydroxy-5-norbornene-2,3-dicarboximide was reacted with epichlorohydrin to give the epoxide **39**. The treatment of **39** with an excess of *i*PrNH_2_ or *t*BuNH_2_ afforded amino alcohol **40** or **41**. Oxyamines **42** and **43** were obtained by aminolysis with NH_3_-MeOH 7 N of **40** or **41**.

The condensation between derivative **42** or **43** and *o*-, *m*- or *p*-hydroxybenzaldehyde afforded the desired amino alcohols **14**–**16**, purified by crystallisation as organic salts.

All the final compounds have been achieved as organic salt oximes presenting *E* configuration. The configuration of the C=N bound for compounds **1**, **2**, **4**–**8**, **12** and **13** was assigned by analogy considering the *E* configuration of starting oximes **17**, **18**, **20–22**, **25**–**27**. As previously reported, the configuration of *E*-oximes **17, 18, 20**–**22, 25**–**27** was stable under the following reaction conditions.

The *E* configuration of amino alcohols **14**–**16** was conferred by comparison between the ^1^H signals of the iminic protons of compounds **14**–**16** with the same signals of final compounds **1**, **2**, **4**–**8**, **12** and **13**. The chemical shift of iminic proton signal resulted around δ 8.05–8.50 ppm for all derivatives as usually reported for oximes with *E* configuration.

### 3.2. Carbonic anhydrase activation

Amino alcohols **1–16** were here assayed for their activating properties of 4 catalytically active and physiologically relevant hCA isoforms expressed in human brain, that are: the cytosolic hCA I, II, and VII, and the membrane associated hCA IV^58^. In the CNS context, hCA I is expressed in the motor neurons in human spinal cord^59^. The physiologically dominant isoform hCA II is located both in the choroid plexus, and in oligodendrocytes, myelinated tracts, astrocytes and myelin sheaths in the vertebrates brain^60^. hCA IV is located on the luminal surface of cerebral capillaries, associated with the blood–brain barrier, and expressed in layers III and VI in the cortex, thalamus and hippocampus^60^^,^^61^. CA VII is expressed in the cortex, hippocampus and thalamus^62^^,^^63^. CA VII might be considered a brain-associated CA as it is predominantly expressed in the brain, and absent in most other tissues. CA VII is also considered a key molecule in age-dependent neuronal pH regulation.

The CA activation data of these 4 isoforms with amino alcohols **1–16** and histamine as standard CAA are shown in Table 1. The following structure–activity relationship (SAR) can be worked out:

**Table 1.** Activation data of human CA isoforms I, II, IV and VII with amino alcohols **1–16** and histamine as reference CAA by a stopped flow CO_2_ hydrase assay^34^. 
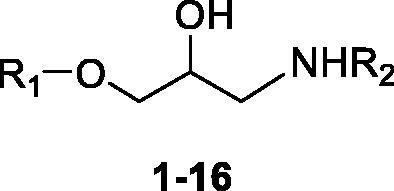

Compound	R_1_	R_2_	Ref	K_A_ (µM)^a^			
				hCA I	hCA II	hCA IV	hCA VII
**1**		*t*Bu	6	0.94	0.76	1.42	0.89
**2**		*t*Bu	1,6	4.25	0.98	6.13	0.95
**3**		*t*Bu	6,7	7.54	7.19	5.94	1.03
**4**		*t*Bu	1	1.37	6.35	4.20	2.40
**5**		*t*Bu	1	7.10	0.079	6.01	0.42
**6**		*t*Bu	1	1.26	0.31	5.29	0.13
**7**		*t*Bu	―	6.02	2.48	1.01	0.74
**8**		*t*Bu	―	0.92	0.47	7.13	0.24
**9**		*i*Pr	2,6	12.1	2.50	7.73	0.082
**10**		*i*Pr	3,6	8.15	1.94	1.08	0.091
**11**		*i*Pr	6,7	8.14	3.27	12.9	8.86
**12**		*i*Pr	4	9.20	4.02	3.24	1.02
**13**		*i*Pr	5	4.57	5.90	3.01	0.51
**14**		*i*Pr	1	n.d	n.d	n.d	n.d
**15**		*t*Bu	1	n.d	n.d	n.d	n.d
**16**		*i*Pr	1	n.d	n.d	n.d	n.d
**Histamine**	–			2.1	125	25.3	37.5

^a^From three different assays (errors within ± 10% of the reported values).

- A special mention should be done for phenol derivatives **14–16** which, uniquely among amino alcohols **1–16**, did not produce any activation of the tested hCAs. In fact, it is worth stressing that CAI properties, rather than as CAAs, are commonly ascribed to the phenolic chemotype. Phenols have been thoroughly reported to anchor to the zinc-bound nucleophile (water molecule or hydroxide ion), that is, by one of the four CA inhibition mechanisms known to date. Surprisingly, neither a significant inhibition was detected by treating CA I, II, IV and VII with phenols **14**–**16**. One could speculate that the CAI efficacy of **14–16** is counterpoised by their CAA action, hindering its detection by the Stopped Flow assay.
- The cytosolic isoform hCA I was moderately activated by all remaining amino alcohols, that are **1–13**, with K_A_ values in the range of 0.92–12.1 µM. The *tert*-butylamino derivatives **1–8** induce a slightly greater activation of CA I than isopropylamines **9–13**. In the **1–8** subset, it can be noted that the *o-* or *p-*substitution of the aromatic ring bearing the oxime ether group lower the K_A_ values **4**, **6**, **8** up to fivefold with respect to **2** (K_A_s of 1.37, 1.26 and 0.92 vs 4.25 µM). In contrast, *m*-substitutions are deleterious for the binding to the target as in **5** and **7** (K_A_s of 7.10 and 6.02 µM). The cyclisation of the oxime ether group to the aromatic ring as in **1** led to the second-best CA I activation after **8** (K_A_s of 0.94 vs 0.92 µM).
- The physiologically most relevant isoform hCA II was activated by most tested amino alcohols more than hCA I (K_A_s in the range 0.079–7.19 µM), except for compounds **4** and **13** (K_A_s of 6.35 vs 5.90 µM). Surprisingly, the *m-*anisole derivative **5** reported a 100-fold enhancement of activation efficacy of hCA II when compared to hCA I (K_A_s of 0.079 vs 7.10 µM). Most remaining *tert*-butylamino derivatives showed submicromolar K_A_ values, whereas all compounds of the subset **9–13** activate hCA II in a low micromolar range (K_A_s in the range 1.94–5.90 µM). All CAAs reported here were more active towards hCA II than histamine, which is a quite weak activator of this isoform with a K_A_ of 125 µM.
- No submicromolar K_A_ values were measured for amino alcohols **1–13** as hCA IV activators. Indeed, all K_A_s are in a rather flat low micromolar range (K_A_s in the range 1.01–12.9 µM), making such a membrane associated isozyme the less activated by the assayed derivatives. Notably, the reference CAA histamine even less activates hCA IV with a K_A_ of 25.3 µM. No significant differences exist between *tert*-butyl and isopropyl derivatives as for the activation of hCA IV.
- The other cytosolic isoform investigated here, hCA VII, was rather potently activated, that is chiefly in a submicromolar range, by most of the compounds reported in this work (K_A_s in the range 0.082–8.86 µM). All derivatives showed much better activation profile than the reference histamine (K_A_ of 37.6 µM) towards hCA VII. Contrariwise to the other hCAs tested, the most efficient CAAs are the isopropylamino derivatives **9** and **10**, ethers of secondary oxime with an aliphatic pendant showing medium nanomolar K_A_s values of 82 and 91 nM, respectively. Only the oxime ethers of *p-*anisaldehyde and acetone in the isopropylamino and *tert*-butylamino series, respectively, that are compounds **4** and **11**, show K_A_s steadily in a low micromolar range. As this isoform is one of the most widely spread in the brain, and probably involved in crucial metabolic/pH regulation processes, these results are of interest in the search of more effective CA VII activators than the currently available such derivatives.

## 4. Conclusions

In the present study, we described the synthesis and CA activating effects of a series of oxime ether-based amino alcohols towards four hCA isoforms expressed in human brain, that are CA I, II, IV and VII. Except for the phenolic compounds **14–16**, all amino alcohol derivatives from this study induce a consistent activation of the tested CAs, with K_A_s spanning from a low micromolar to a medium nanomolar range. Specifically, hCA II and VII, probable main CA targets when CNS diseases are concerned,^58^ are most potently activated by these oxime ether derivatives. With K_A_s of 79 and 420 nM towards hCA II and VII, respectively and K_A_s for CA I and IV settling in a range 10- to 100-fold higher, derivative **5** from the *tert*-butylamines series turned out as the most potent and selective hCA II activator of the study. On the other hand, a multitude of selective hCA VII activators were identified. Specifically, a mention should be done for derivatives **9** and **10**, from the isopropylamines series, showing a 10- to 100-fold selective hCA VII activation profiles with respect to all other assayed CAs. In this second youth period for CAAs, innovative pharmacological studies made these lately neglected agents to draw attention in the memory therapy and cognitive neurodegenerative disorders, as well as in therapeutic areas, such as phobias, obsessive-compulsive disorder, generalised anxiety, and post-traumatic stress disorders^1^^,^^20^. As hCA VII is a key isoform involved in brain metabolism, the here identified potent and selective hCA VII activators may be considered of interest for investigations of possible therapeutic applications or as lead compounds in search of more potent and selective CAAs. This work might bring new lights on the intricate relationship between CA activation and brain physiology.
